# XPS Modeling of Immobilized Recombinant Angiogenin and Apoliprotein A1 on Biodegradable Nanofibers

**DOI:** 10.3390/nano10050879

**Published:** 2020-05-02

**Authors:** Anton Manakhov, Elizaveta Permyakova, Sergey Ershov, Svetlana Miroshnichenko, Mariya Pykhtina, Anatoly Beklemishev, Andrey Kovalskii, Anastasiya Solovieva

**Affiliations:** 1 Research Institute of Clinical and Experimental Lymphology—Branch of the ICG SB RAS, 2 Timakova str., 630060 Novosibirsk, Russia; permyakova.elizaveta@gmail.com (E.P.); svmiro@yandex.ru (S.M.); pykhtina_maria@mail.ru (M.P.); beklem@niibch.ru (A.B.); solovevaao@gmail.com (A.S.); 2 Laboratory for the Physics of Advanced Materials (LPM), Department of Physics and Materials Science, University of Luxembourg, L-1511 Luxembourg, Luxembourg; sergey.ershov@uni.lu; 3 Institute of Biochemistry, FRC FTM 2 Timakova str., 630117 Novosibirsk, Russia; 4 National University of Science and Technology “MISiS”, Leninsky pr. 4, 119049 Moscow, Russia; andreykovalskii@gmail.com

**Keywords:** biotechnology, nanofibers, plasma, polymers, X-ray photoelectron spectroscopy, angiogenin

## Abstract

The immobilization of viable proteins is an important step in engineering efficient scaffolds for regenerative medicine. For example, angiogenin, a vascular growth factor, can be considered a neurotrophic factor, influencing the neurogenesis, viability, and migration of neurons. Angiogenin shows an exceptional combination of angiogenic, neurotrophic, neuroprotective, antibacterial, and antioxidant activities. Therefore, this protein is a promising molecule that can be immobilized on carriers used for tissue engineering, particularly for diseases that are complicated by neurotrophic and vascular disorders. Another highly important and viable protein is apoliprotein A1. Nevertheless, the immobilization of these proteins onto promising biodegradable nanofibers has not been tested before. In this work, we carefully studied the immobilization of human recombinant angiogenin and apoliprotein A1 onto plasma-coated nanofibers. We developed a new methodology for the quantification of the protein density of these proteins using X-ray photoelectron spectroscopy (XPS) and modeled the XPS data for angiogenin and apoliprotein A1 (Apo-A1). These findings were also confirmed by the analysis of immobilized Apo-A1 using fluorescent microscopy. The presented methodology was validated by the analysis of fibronectin on the surface of plasma-coated poly(ε-caprolactone) (PCL) nanofibers. This methodology can be expanded for other proteins and it should help to quantify the density of proteins on surfaces using routine XPS data treatment.

## 1. Introduction

The synthesis of new nanomaterials for biomedical applications requires careful tuning of the surface topography, structure, and chemical composition [1,2]. Recently, it was found that the immobilization of an ensemble of viable biomolecules, e.g., platelet-rich plasma (PRP) or different growth factors, may induce superior bioactivity of polymeric nanomaterials [3]. Moreover, even single-protein immobilization (e.g., fibronectin, apoliprotein, etc.) was found to be responsible for the enhanced bioactivity on the surfaces.

The need for a weak (non-covalent), strong (covalent), or complex bonding of protein molecules to scaffolds depends on the intended use of the biomaterial. For the purposes of cell therapy, the bonding strength of proteins to the matrix ensures good survival and functional activity of cells compared to non-covalent bonding, which has a more short-term effect [3].

These scaffolds can be used as cell transplants. The strength of the bonding should be adjusted accordingly for different proteins. For example, covalently immobilized fibronectin is better protected (as compared to non-covalently immobilized fibronectin) from proteolysis in places with rapidly disintegrating extracellular matrix (chronic wounds), and thus may constitute the necessary structural base for cell regeneration. For the delivery of biologically active substances, the weak bonding will provide a quick release of proteins into the microenvironment, which in turn contains reactive oxygen species (ROS), the enzymes that destroy and modify the proteins [4,5]. 

Angiogenin (ANG), also known as ribonuclease 5, is a single-chain protein with a mass of 14.4 kDa, composed of 123 amino acids. Angiogenin is a vascular growth factor that is similar to vascular epidermal growth factor (VEGF). Recently, it was shown that angiogenin can be considered as a neurotrophic factor influencing the neurogenesis, viability, and migration of neurons [6]. It plays an important role in protection of neurons against oxidative damages by affecting the Nrf2/ARE (NF-E2-related factor 2) antioxidant pathways [7]. It also takes part in innate immunity and inflammatory diseases. ANG causes a decrease in the pro-inflammatory cytokines IL-1β, IL-6, and IL-8, while increasing the anti-inflammatory cytokines IL-4 and IL-10 [8]. Due to its RNase activity, ANG demonstrates antibacterial properties [9] and it has been reported that it can be considered as a microbial recognition protein related to the innate immunity [10].

Moreover, angiogenin has a combination of factors that are necessary for the restoration and protection of organs and tissues. An exceptional combination of angiogenic, neurotrophic, neuroprotective, antibacterial, and antioxidant activities makes this protein a promising molecule for the immobilization of carriers, with the goal of increasing regenerative properties, particularly for diseases that are complicated by neurotrophic and vascular disorders. Therefore, the investigation of the immobilization of angiogenin (ANG) in biodegradable nanofibers is of high interest for material science and biomedicine.

Apoproptein A1 (Apo-A1) is the main component of high-density lipoproteins (HDL). In addition to its main function of binding and exchanging lipids, it is also able to neutralize single-electron free radical oxidizers. The amino acids located at positions 112 and 148 of Apo-A1 participate in redox reactions, decreasing the amounts of toxic lipid oxidation products [11]. 

The effect of this protein on the functional activity of cells has also been reported. It was shown that Apo-A1 exerts a protective effect on the endothelial cells, inhibiting apoptosis and increasing cell proliferation [12]. It is also involved in anti-inflammatory actions of the body, affecting both innate and acquired immunity [13,14]. 

The antiviral activity of Apo-A1 has been shown in [15]. The protein forms hydrophobic interactions with the extracellular matrix (ECM) through binding to fibronectin and collagen I [16]. A high content of alpha helices (due to the amphipathic structure) provides the main functions of Apo-A1 [17,18]. 

Owing to its anti-inflammatory, antioxidant, and antiviral properties, as well as its ability to bind to the main protein components of the extracellular matrix, this protein is of high interest for scaffolding in regenerative medicine. Hence, it is highly important to investigate the bonding of such a protein to the surfaces of materials intended for tissue engineering and to carefully analyze the protein–surface interactions.

Currently, there is lack of understanding regarding the chemical linkages between the proteins and surfaces. The majority of the surface analyses dedicated to the investigation of protein–surface interactions are focused on the analysis of immobilized fibronectin [4,19]. The methodology of fibronectin detection is quite complex and requires antibody staining. In the majority of works, the immobilization of fibronectin is confirmed only qualitatively by certain changes in the atomic concentrations, e.g., an increase of nitrogen or by small changes in the carbon environment derived from the X-ray photoelectron spectroscopy (XPS) C1s curve fitting [20]. No quantitative calculation methodology could be found.

The situation regarding the available research data is even more complicated when considering the immobilization of less-abundant proteins (e.g., Apo-A1 or ANG). For example, to our knowledge, surfaces with immobilized Apo-A1 or ANG have never been analyzed by XPS or IR spectroscopy. 

The challenge becomes much greater in real application conditions, where the structure and chemical composition of the substrate might be very complex. Recently, nanofibrous mats have become very popular substrates for tissue engineering due to their structure being similar to that of the extracellular matrix (ECM) [21,22,23]. Nonetheless, their surface often requires modification by plasma treatment in order to graft the active surface groups, e.g., COOH, NH2, and OH. These surface groups are subsequently used for the chemical immobilization of proteins [24].

The detection of proteins using antibody staining and fluorescence confocal imaging is a rather expensive and time-consuming process, which is only qualitative, or at best, semi-quantitative. The development of a quantification procedure for the XPS data of proteins immobilized on the surface would, therefore, be very useful. Such a method could also be used as a complimentary tool in order to better understand the complex nature of the surface–protein interactions. 

In this work, the immobilization of apoliprotein A1 (Apo) and recombinant angiogenin (ANG) on biodegradable nanofibers is investigated by XPS, FT-IR, and SEM analyses, while a special XPS curve fitting procedure involving the simulation of protein spectra is suggested as a promising method for the quantification of immobilized proteins.

## 2. Materials and Methods 

### 2.1. Electrospining of Nanofibers

The electrospun nanofibers were prepared by the electrospinning of a 9 wt% solution of poly(ε-caprolactone) (PCL; 80000 g/mol). The processing of the sample can be found elsewhere [25]. Briefly, the granulated PCL was dissolved in a mixture of acetic acid (99%) and formic acid (98%). All compounds were purchased from Sigma-Aldrich (Darmstadt, Germany). The weight ratio of acetic acid (AA) to formic acid (FA) was 2:1. The PCL solutions in AA and FA were stirred at 25 °C over 24 h. The PCL solution was electrospun with a 20-cm long wired electrode using a Nanospider™ NSLAB 500 machine (ELMARCO, Liberec, Czech Republic). The applied voltage was 50 kV. The distance between the electrodes was set to 100 mm. The as-prepared and non-treated PCL nanofibers are referred to as PCL-ref throughout the text. 

### 2.2. Deposition of COOH Plasma Layer

The COOH plasma polymer layers were deposited using the UVN-2M vacuum system equipped with rotary and oil diffusion pumps. The residual pressure of the reactor was below 103 Pa. The plasma was ignited using a Cito 1310-ACNA-N37A-FF radio frequency (RF) power supply (Comet, Flamatt, Switzerland) connected to a RFPG-128 disk generator (beams and plasma) installed in the vacuum chamber. The duty cycle and the RF power were set to 5% and 500 W, respectively. 

CO_2_ (99.995%), Ar (99.998%), and C_2_H_4_ (99.95%) were fed into the vacuum chamber. The flows of the gases were controlled using a 647C Multi-Gas Controller (MKST, Newport, RI, USA). The flow rates of Ar, CO_2_, and C2H4 were set to 50, 16.2, and 6.2 sccm, respectively. The pressure in the chamber was measured by a VMB-14 unit (Tokamak Company, Dubna, Russia) and D395-90-000 BOC Edwards controllers. The distance between the RF electrode and the substrate was set to 8 cm. The deposition time was 15 min, which led to a growth of ~100-nm thick plasma coatings. The plasma-coated PCL nanofibers are referred to as PCL-COOH throughout the text.

### 2.3. Synthesis of Proteins

The apoliprotein A1 was isolated from healthy blood donors. Briefly, plasma of blood samples underwent isodensity ultracentrifugation (Optima L-90K, Beckman-Coulter, Indianapolis, IN, USA) in KBr solutions at 105,000× *g* to isolate high-density lipoproteins (HDL). Stages of subsequent delipidation of the HDL fraction and purification of ApoA-I were carried out according to [26]. The purity of the final preparation was checked by electrophoresis in 12.5% PAAG according to the Laemmli method, using a set of protein markers (Sibenzyme 10-250 kDa). The purity of apoA-I was at least 95%

Human recombinant angiogenin (hrAng) was obtained using expression of a synthetic gene of this protein in *Escherichia coli* strain BL21 (DE3), as described in [27]. Based on the sequences of two IgG-binding domains of *Staphylococcus aureus (S. aureus)* protein A (Z-region) in the plasmid vector pEZZ18A (GenBank # M74186) and the amino acid sequences of mature human angiogenin (GenBank # AAA51678; # AAL67710; # AAL67712), the primary structure of the corresponding chimeric gene containing the codon composition optimal for expression in *E. coli* cells was calculated. 

The constructed *E. coli* BL21 (DE3)/pJZZ-A strain produces a recombinant chimeric angiogenin (hrANG). The hrANG contains an amino acid sequence of an artificial leader, with 8 amino acid residues in the N-terminal region, followed by two IgG-binding domains (ZZ) of *Staphylococcus aureus* protein A, while the sequence of mature human angiogenin is in the C-terminal region. An amino acid sequence diagram of hrANG (ZZ-hAngiogenin) and its spatial structure are shown in Figure S1 (see supporting information). The molecular weight of hrANG was 28 kDa.

### 2.4. Immobilization of Proteins

Prior to immobilization of proteins, all samples were sterilized under UV for 45 min. At first, the adsorption of human recombinant angiogenin and apoprotein A1 by PCL-ref was investigated. The PCL-ref was immersed in the ANG solution for 15 min at 25 °C, and then it was thoroughly washed with phosphate buffer saline (PBS). The same procedure was repeated for apoprotein A1. The samples with immobilized hrAng and apoliprotein A1 were denoted as PCL-ANG and PCL-Apo, respectively. In order to achieve the covalent bonding of these proteins to the plasma-treated PCL-COOH surface, the latter was immersed in the 1-Ethyl-3-(3-dimethylaminopropyl) carbodiimide (EDC) (98% Sigma Aldrich, Darmstadt, Germany) solution in water (2 mg/mL) for 15 min at room temperature. The samples were carefully washed by PBS and then incubated with hrAng, apoprotein A1, or fibronectin (FN) at 25 °C for 15 min. After reaction, the samples were once more thoroughly washed with PBS (in order to remove all the adsorbed proteins and to keep only the covalently bound proteins on the surface). These samples were denoted with respect to the immobilized protein as PCL-COOH-ANG, PCL-COOH-Apo, and PCL-COOH-FN, respectively. Note that for the sake of simplicity, apoliprotein A1 and hrANG are denoted in samples as Apo and ANG, respectively. The immobilization of fibronectin (Applichem, USA) onto PCL-COOH layer was performed in the same way and the resulted sample was denoted as PCL-COOH-FN.

### 2.5. Measurement of Protein Concentration

The concentration of apoliprotein A1 was measured by the fluorescence method using a Typhoon FLA 9500 Imager scanner (Lissone, Italy). First, the dependence of the fluorescence signal as a function of the Apo-A1 concentration in PBS was measured. Then, the fluorescence signal of PBS was subtracted. The calibration curve is presented in Figure S3 of the Supporting Information. The fluorescence signal from 40 µL of PBS (used for the washing of PCL-Apo or PCL-COOH-Apo) was measured. The fluorescence signals from PBS used for the soaking of samples after the immersion times of 20 min, 48 h, and 144 h at 37 °C and relative humidity of 95% were recorded. The concentration of apoliprotein A1 in each solution was quantified using the calibration curve and Equation S1 (see Supporting Information). The total mass of the immobilized apoliprotein A1 was calculated as a sum of masses of apoliprotein A1 (40 µL × concentration) after each washing with the PBS solution.

### 2.6. Characterization of Samples

The microstructures of the nanofibers and the deposited plasma polymers were studied by scanning electron microscopy (SEM) using a JSM F7600 (Jeol Ltd., Tokyo, Japan) device. The SEM micrographs were obtained with an accelerating voltage of 2 kV and a scan time of 1 min. In order to compensate for the surface charging, the samples were coated with a ~5 nm thick Pt layer by magnetron sputtering.

The chemical composition of the sample surfaces was determined by the X-ray photoelectron spectroscopy (XPS) using an Axis Supra spectrometer (Kratos Analytical, Manchester, UK) equipped with a monochromatic Al Kα X-ray source. The maximum lateral resolution of the analyzed area was 0.7 mm. The spectra were fitted using the CasaXPS software after subtracting the Shirley-type background. The binding energies (BE) for all carbon and nitrogen environments were taken from the literature [24,25,28]. The BE scale was calibrated by setting the CH_x_ component at 285 eV.

The infrared spectra ranging from 370 to 4000 cm^−1^ were measured using a Fourier transform infrared (FT-IR) spectrophotometer (Bruker Vertex 80v, Ettlingen, Germany) in the attenuated total reflectance mode (ATR-FT-IR).

## 3. Results

### 3.1. Immobilization of Proteins onto PCL-ref by Adsorption

**Figure 1:**
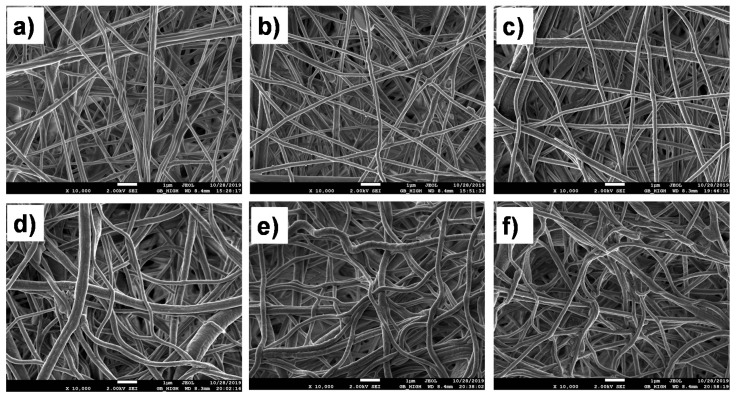
SEM micrographs of PCL-ref (**a**), PCL-Apo (**b**), PCL-ANG (**c**), PCL-COOH (**d**), PCL-COOH-Apo (**e**) and PCL-COOH-ANG (**f**). The size of the bar is 1 µm.

The SEM micrographs of electrospun nanofibers (without plasma treatment) before and after immobilization of proteins are presented in Figure 1a–c. The fiber diameter was around 250 nm. It can be clearly seen that the microstructure of the nanofibrous mats was not affected by the procedure.

The XPS analysis revealed that the surface of PCL-ref is composed entirely of carbon and oxygen. The immobilization of hrANG and apoliprotein A1 onto PCL-ref caused noticeable changes in the surface chemistry. While the immobilization of apoliprotein A1 (PCL-Apo) was evidenced by the determination of 2.2 at.% of nitrogen and by a decrease of the O/C ratio from 0.35 to 0.29, the immobilization of ANG (PCL-ANG) only led to a decrease of the O/C ratio from 0.35 to 0.28. One might be tempted to think that the absence of the nitrogen implies the unsuccessful immobilization of ANG. However, one should also remember that the limit of detection for nitrogen by XPS is about 0.3–0.4 at.% and that the nitrogen content on the surface of PCL-ANG could be below this limit.

**Figure 2:**
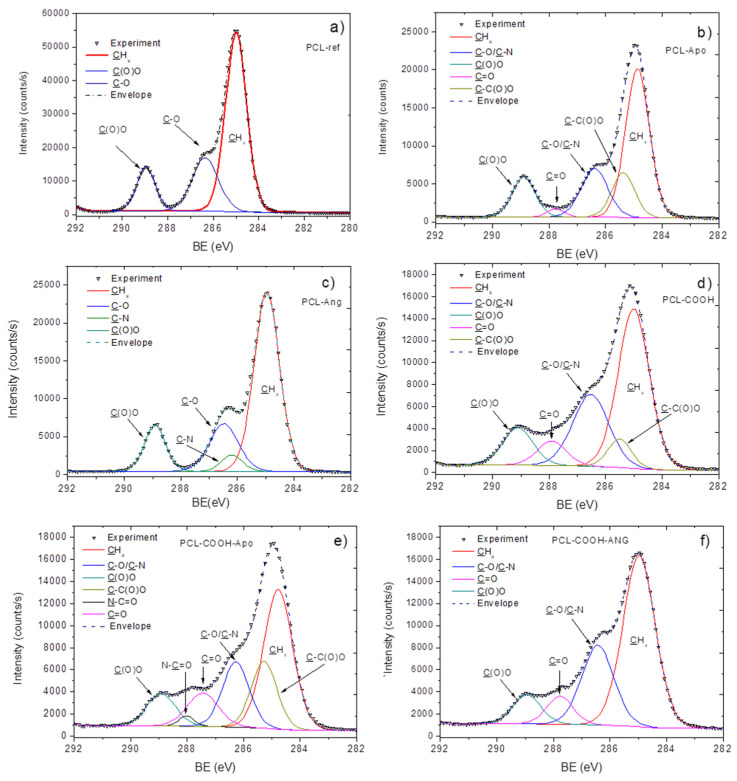
The XPS C1s curve fitting of PCL-ref (**a**), PCL-Apo (**b**), PCL-ANG (**c**), PCL-COOH (**d**), PCL-COOH-Apo (**e**), PCL-COOH-ANG (**f**).

**Figure 3:**
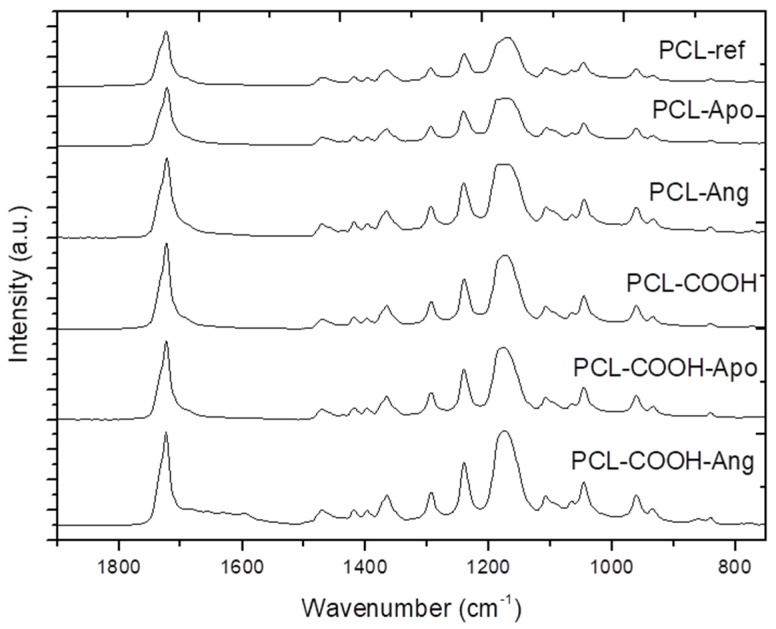
The FT-IR spectra of the PCL nanofibers.

In order to more deeply investigate the interaction of proteins with the PCL-ref nanofibers, the FT-IR and XPS high-resolution spectra of samples with immobilized proteins were studied. The XPS C1s spectra of PCL-ref (Figure 2a), PCL-ANG (Figure 2b), and PCL-Apo (Figure 2c) were quite different and they were fitted with different models. PCL-Apo was fitted with a sum of five components: CH_x_ (BE = 285 eV), C-C(O)O(BE = 285.5 eV) C–O/C–N (BE = 286.5 eV), C=O/N–C=O (BE = 287.7 eV), and C(O)O (BE = 288.9 eV). The PCL-ANG was fitted with the sum of four components: CH_x_ (BE = 285 eV), C–N (BE = 286.2 eV), C–O (BE = 286.5 eV), and C(O)O (BE = 288.9 eV). The full width at half maximum (FWHM) value for all components was set to 1.1 ± 0.1 eV. The XPS C1s spectra of PCL-Apo and PCL-ANG are presented in Figure 2b,c. Although certain changes in carbon environments are evident, it is not possible to quantify the density of the immobilized proteins. The comparison of FT-IR spectra of PCL-ref, PCL-Apo, and PCL-ANG (Figure 3) did not reveal significant differences after the immobilization. As for the nitrogen environment, the XPS N1s curve fitting of all samples revealed that nitrogen consists mainly of the amide environment (N–C=O), because the N1s signal can be fitted with only one component at ~399.9 eV, as shown in Figure S3. Indeed, for the proteins containing many peptide bonds, this observation is very consistent with expectations, although it is not very informative. On the other hand, the semi-quantitative information can still be extracted from the data via a careful analysis of the XPS data, which will be presented in Section 3.4, opening a door leading to a new series of protein–surface interaction investigations

One might consider that the adsorption of proteins by the unmodified PCL-ref is rather insufficient and that XPS and FT-IR, as a consequence, do not have enough sensitivity to detect the changes after immobilization. In order to facilitate the covalent bonding of proteins to nanofibers, the investigation of the immobilization of ANG and Apo-A1 onto the plasma-treated nanofibers was carried out, with the results reported below.

### 3.2. Immobilization of Proteins onto PCL-COOH by Covalent Linkage

The SEM investigation (Figure 1d–f) revealed that the immobilization of proteins onto PCL-COOH led to minor changes in the morphology of the PCL nanofibers; the fibers became more curved and their density increased. The immobilization of proteins onto PCL-COOH was evidenced by both XPS and FT-IR analyses. As shown in Table 1, both PCL-COOH-Apo and PCL-COOH-ANG exhibited higher nitrogen concentrations as compared to PCL-Apo and PCL-ANG, respectively. At the same time, for both plasma-treated samples, the concentration of nitrogen did not exceed 3 at.%. The high-resolution C1s spectrum of PCL-ANG was fitted with the sum of four components: CH_x_ (BE = 285 eV), C–O/C–N (BE = 286.4 eV), C=O (BE = 287.8 eV), and C(O)O (BE = 289.0 eV). The PCL-Apo was fitted with the sum of five components: CHx (BE = 285 eV), C–N/C–O (BE = 286.5 eV), C=O (BE = 287.4 eV), N–C=O (BE = 287.9 eV), and C(O)O (BE = 289.0 eV). In the case of PCL-COOH-Apo, an additional component attributed to the amide group (N–C=O) was required for sufficient quality of fitting. The BE values of C=O and N–C=O were too different, and as a result it was not possible to merge these two moieties into one component. C=O most likely represents ketone or acetal groups from the plasma coating, whereas N–C=O is related to the amide group of the apoliprotein A1.

### 3.3. Analysis of Immobilized Proteins by the Fluorescence Microscopy

The amount of the immobilized proteins on the surface of PCL-ref and PCL-COOH was investigated by analysis of the concentration of Apo-A1 in the liquid phase after the soaking of PCL-Apo and PCL-COOH-Apo in PBS. The analysis of the concentration of Apo-A1 was performed for both samples using the same sample size and the same time intervals. It was found that the surface of PCL-APO released all the adsorbed proteins after 20 min of soaking in PBS, whereas the surface of PCL-COOH-APO released proteins for more than a week. The masses of the immobilized Apo-A1 on PCL-ref and PCL-COOH were 12.4 and 20.6 μg/cm2, respectively. The covalent immobilization led to a long-term linkage of proteins to the surfaces of nanofibers and to a larger amount (by more than 60%) of the introduced Apo-A1. 

### 3.4. Modeling of the XPS C1s Spectra of Proteins and Layers

**Figure 4:**
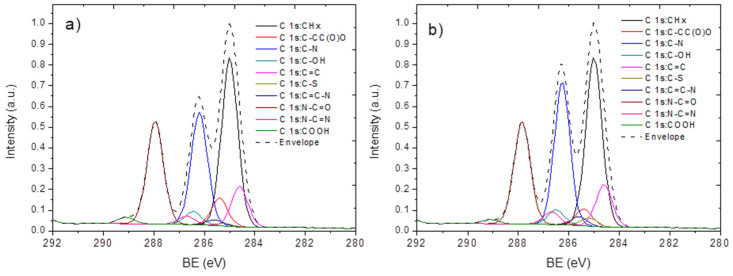
The calculated XPS C1s spectra of apoliprotein A1 (**a**) and human recombinant angiogenin (**b**).

In order to more deeply investigate the interaction of proteins with PCL-ref and PCL-COOH, it would be useful to simulate the XPS C1s spectra of Apo-A1 and angiogenin. The amino acid sequences of both proteins were taken from the GenBank database. The accession number for Apo-A1 is AAB59514, and for the ZZ region and human recombinant angiogenin of chimeric protein the accession numbers are M74186 and AAL67710, respectively. For every amino acid, each carbon atom can be represented as having the following environments: C=C, CH*_x_*, C=C–N, C–C(O)O, C–OH, C–N, C=N, N–C=O, C-S, and COOH. The BE values of these environments can be found in the literature [29]. By calculating the sum of all types of atoms and then normalizing this to the number of carbon atoms from each environment, we obtained the concentration of each type of carbon atom in the representative spectra of Apo-A1 and ANG. The raw data and the calculated concentration of each carbon environment are reported in Tables S1 and S2 of the Supporting Information. On the basis of the calculated concentration of each carbon environment and the binding energy data from the literature, we calculated the XPS C1s spectra of Apo-A1 and ANG (Figure 4). Such a model can be used for the special fitting of the XPS data in order to quantify the concentration of immobilized proteins.

The direct comparison of the simulated spectra of proteins with the spectra of PCL-ANG, PCL-Apo, PCL-COOH-ANG, and PCL-COOH-Apo led to several important conclusions. Firstly, it is clear that the structures of the XPS C1s spectra of proteins and the real experimental data are very different, i.e., the density of immobilized proteins is quite low. Secondly, the characteristic features of the protein spectra can be used for the special curve fitting, where instead of the classical component decomposition we may use the sum of two spectra—the simulated spectrum of a protein and the real XPS C1s signal of PCL-COOH. This procedure requires the normalization of the spectra; therefore, the signals for PCL-COOH and the simulated spectrum were both normalized to the maximum signal. Then, the resulting spectrum was calculated according to Equation (1):

(1) $$S_{res}=S_{exp}\times \left(1-x\right)+S_{Protein}\times x$$

where S_res_ is the signal of the resulting XPS C1s spectrum, S_exp_ is the normalized signal of the experimentally measured PCL-ref or PCL-COOH, S_protein_ is the signal of the simulated spectrum of apoprotein A1 or human recombinant angiogenin, and *x* is the concentration of protein on the surface.

**Figure 5:**
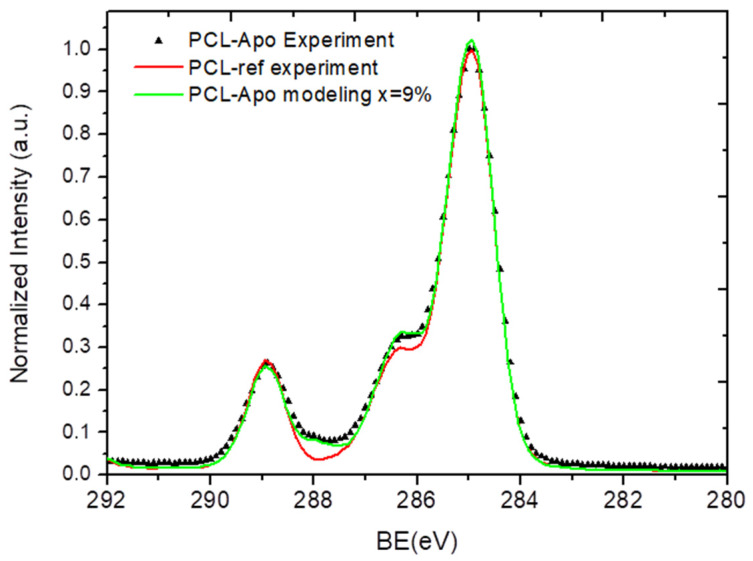
A comparison of the XPS C1s spectra: the experimentally measured PCL-Apo (PCL-Apo-Experiment), PCL-ref, and calculated PCL-Apo (PCL-Apo-modeling).

**Figure 6:**
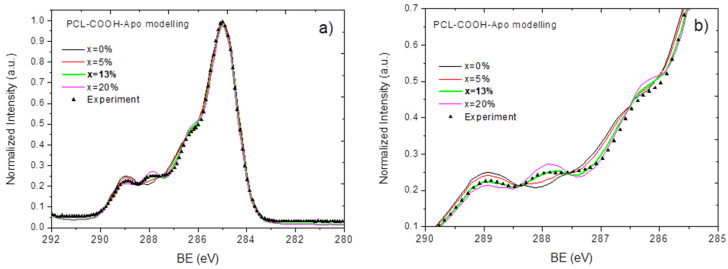
A comparison of the XPS C1s signals for PCL-COOH-Apo, which were measured experimentally and modeled with the different concentrations of protein (x) on the surface. An overview (**a**) and an enlarged image of the spectrum (**b**) are given to better show the effect of x on the similarity of the calculated spectrum to the experimental data.

By varying x and targeting the best convergence (similarity) of the calculated S_res_ to the experimentally measured spectra of PCL-Apo-A1 and PCL-COOH-Apo, it was possible to determine the concertation of protein on the surface of PCL-Apo or PCL-COOH-Apo (see Figure 5 and Figure 6).

From Figure 5 it can be seen that at x = 9%, the modeled and the experimentally measured spectra of PCL-Apo-A1 are identical. The spectrum of PCL-COOH-Apo-A1 was modeled and is presented in Figure 6. By varying x from 0% to 20%, the best convergence between the modeled and the experimentally measured spectra was found for x = 13%. Hence, the covalent immobilization of APO-A1 onto PCL-COOH led to a 44% increase in the protein surface density as compared to the non-covalent adsorption in the case of PCL-APO. This increase of the protein surface density is consistent with the higher concentration of APO-A1 on the surface of PCL-COOH-APO measured by the fluoresce microscopy, as presented in Section 3.3.

**Figure 7:**
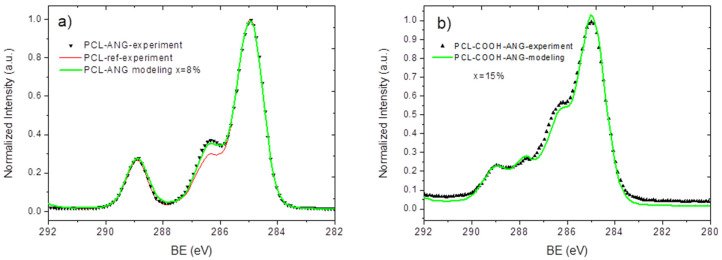
A comparison of the XPS C1s signals of PCL-ANG (**a**) and PCL-COOH-ANG (**b**), which were measured experimentally and modeled using Equation (1).

A similar procedure was applied for the modeling of PCL-COOH-ANG. The interaction of angiogenin with the PCL-ref and PCL-COOH surfaces seemed to be more complex, leading to significant changes in the expected (calculated) and real spectra. The modeling of PCL-ANG led to relatively good convergence at x = 8% (Figure 7a). However, for PCL-COOH-ANG (Figure 7b), the convergence of the modeled and experimentally measured spectra was less evident. The best similarity was achieved with x = 15%.

**Figure 8:**
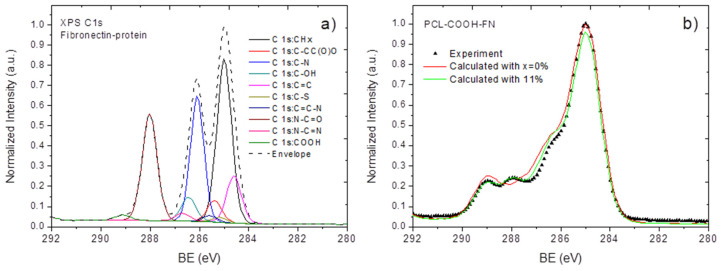
The XPS C1s spectra of the simulated fibronectin (**a**) and a comparison of the experimentally measured and calculated PCL-COOH-FN (**b**).

In order to confirm the applicability of the proposed methodology for modeling of protein spectra and the quantification of the immobilized proteins with the XPS data, we decided to apply our methodology towards the most thoroughly analyzed protein, namely fibronectin (FN). The spectrum of fibronectin was calculated using the aforementioned methodology and is presented in Figure 8a. The modeled FN and the experimentally measured PCL-COOH-FN spectra are shown in Figure 8b.

From Figure 8b, it can be seen that the modeled spectrum with x = 11% led to relatively good convergence with the experimental data. Next, we compare this estimated surface concentration of FN with the concentration estimated from the elemental composition of PCL-COOH-FN using the elemental composition of FN according to Equation (2).

(2) $$Concentration=\frac{Nitrogen\left(experiment\right)}{Carbon\left(experiment\right)}\times \frac{Carbon\left(Protein\right)}{Nitrogen\left(Protein\right)}\times 100\%$$

The carbon/nitrogen ratio in the protein structure obtained from the amino acid sequence of FN is equal to 3.58, whereas the nitrogen/carbon ratio in PCL-COOH-FN is 0.08. The estimated concentration equals 28%. One important question is the reason for this difference, which is discussed below.

## 4. Discussion

Our methodology for investigation of protein immobilization by adsorption or covalent immobilization was validated by experiments with three different viable proteins. For apoliprotein A1 and fibronectin, the methodology showed very good convergence with experimental data, while the immobilization of human recombinant angiogenin was more complicated. The immobilization of proteins onto a simple, well-defined surface (e.g., PCL-ref) is a rather complex process with a few specific aspects. As presented before, although the immobilization of angiogenin onto PCL-ref resulted in an undetectable (by XPS) amount of nitrogen on the surface, the XPS C1s spectra implied the presence of this protein on the sample’s surface. By using the proposed strategy for the quantification of proteins, it was found that 8% and 15% of the surfaces of PCL-ANG and PCL-COOH-ANG were covered with ANG, respectively, although the detected nitrogen concentration was very low. The explanation for this contradiction can be linked to several effects. First, there are differences in structure and size—angiogenin is smaller than apoliprotein A1. Consequently, at the XPS analysis depth (3–6 nm) there would be a smaller protein/PCL ratio. Secondly, due to the higher kinetic energy of the C1s electrons, their escaping depth is bigger than that of the N1s electrons. This could explain possible differences in the determination of C-N moieties from the C1s spectrum and N moieties from the N1s spectrum, and different concentrations of proteins estimated from the atomic composition and from the XPS C1s spectrum fitting.

Finally, due to a small N/C ratio in ANG, the detection of nitrogen, which is related to the protein structure, becomes very challenging, especially with low protein coverage. By taking into account all of the aforementioned aspects, it was found out that for apoprotein A1, both methods of the detection for protein density (from nitrogen concertation and from the XPS C1s spectra) led to reasonable results, as cross-checked by fluorescence microscopy, whereas for ANG only the XPS C1s special curve fitting allowed the protein to be detected. It is worth noting that for apoliprotein A1, both the PCL-Apo and PCL-COOH-Apo samples were perfectly fitted by the proposed model, while for ANG only the PCL-ANG sample showed a suitable fit. The lower convergence of experimental and simulated data for PCL-COOH-ANG presents a rather complicated problem. The mathematical explanation for the worse convergence of PCL-COOH-ANG is the higher density of the carbon environment of around 286.5 eV in the measured spectrum as compared to the model. As a consequence, with the (further) increase of x, the other parts of the spectra cannot be fitted. The most probable explanation for this effect is a strong interaction of protein domains with the COOH plasma polymerized surface and its effect on the spectrum of the initial plasma polymer layer. At the same time, a reasonable question remains as to why the same effect was not visible at all for PCL-COOH-Apo and was almost not visible for PCL-COOH-FN. 

**Figure 9:**
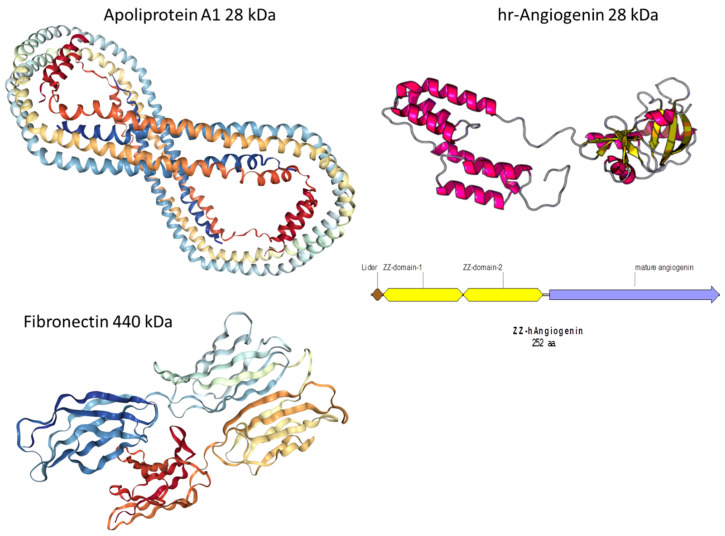
The structure of Fibronectin(FN), human recombinant Angiogenin (hrANG), and Apoliproprotein Apo-A1.

To answer this question, a vast series of analytical experiments would be required. Currently, we can only suggest that due to the specific helical structure of Apo-A1, this protein has a smaller affinity towards the COOH plasma polymer layer (Figure 9). In contrast, ANG exhibits branched domains that are more flexible than α-helices of Apo-A1. Consequently, ANG may have a stronger interaction with the PCL-COOH surface, leading to significant changes in the spectrum of the plasma polymer, and possibly in its own spectrum. 

Our methodology has shown that the XPS data can provide semi-quantitative information on the immobilization of the proteins. However, certain limitations must be taken into account:

- The functional groups of proteins can be hindered by the interaction with the substrate or “neighbors”, and thus the real spectra of pure protein for x = 1 might provide a better convergence;
- The interaction of the COOH groups and EDC requires more careful investigation;
- Further validation of the proposed methodology should be performed.

## 5. Conclusions

In this work, we have demonstrated that the viable proteins apoliprotein A1, human recombinant angiogenin, and fibronectin can be immobilized on PCL nanofibers via covalent bonding or by simple adsorption. It was found that analysis of the proteins on the surfaces of PCL-Apo, PCL-ANG, PCL-COOH-Apo, PCL-COOH-ANG, and PCL-COOH-FN is a very complicated task and requires very careful fitting of the XPS data. The proposed methodology demonstrated that although XPS analysis of PCL-ANG did not show nitrogen, the XPS C1s analysis revealed that 8% of the PCL-ANG surface is covered by human recombinant angiogenin, whereas PCL-COOH-ANG exhibited a protein coverage (x) of 15%. The analysis of PCL-Apo and PCL-COOH-Apo yielded x values of 9% and 13%, respectively. These findings were also confirmed by the analysis of immobilized Apo by fluorescent microscopy. The presented methodology was also validated by the analysis of fibronectin on the surfaces of plasma-coated PCL nanofibers. This methodology can be expanded to other proteins and it would help to quantify the surface density of proteins by routine XPS data treatment. However, it should be noted that the current work represents only the first attempt to perform semi-quantitative estimation of protein density on the surfaces of nanofibers and that further validation tests are foreseen in our future work.

## Figures and Tables

**Table 1:** Composition of samples (in at.%) derived from X-ray photoelectron spectroscopy (XPS) analysis. The traces of sodium, chlorine, and phosphorus were detected but were not taken into account.

Sample Name	[C], at.%	[O], at.%	[N], at.%
PCL-ref	73.9	26.1	0.0
PCL-APO	75.8	22.0	2.2
PCL-ANG	78.0	22.0	0.0
PCL-COOH	72.3	27.5	0.3
PCL-COOH-APO	72.6	24.5	2.9
PCL-COOH-ANG	72.4	26.5	1.1
PCL-COOH-FN	71.3	23.1	5.6

## Supplementary Materials

The following are available online at https://www.mdpi.com/2079-4991/10/5/879/s1: Figure S1: Scheme of a chimeric protein containing two Z-regions of protein A from Staphylococcus aureus and the amino acid sequence of mature human angiogenin, Figure S2: XPS N1s curve fitting of PCL-Apo(a), PCL-COOH-Apo(b), PCL-COOH-ANG(c) and PCL-COOH-FN (d), Table S1: Calculation of the functional groups in the Apo-A1 using its chemical structure, Table S2: Calculation of the functional groups in the Angiogenin using its chemical structure, Figure S3: Calibration curve for APO-A1 concentration measurement.
