# Supplementary material for: XPS Modeling of Immobilized Recombinant Angiogenin and Apoliprotein A1 on Biodegradable Nanofibers
Source: Nanomaterials (Basel). 2020 May 2;10(5):879. doi: 10.3390/nano10050879 (PMC7279301; doi:10.3390/nano10050879)
Supplement: Supplementary file 1 [file nanomaterials-10-00879-s001.pdf]

Supporting Information

XPS modelling of immobilized recombinant Angiogenin and Apoliprotein A1 on biodegradable nanofibers.

Anton Manakhov[1*] , Elizaveta Permyakova[1], Sergey Ershov[2], Svetlana Miroshnichenko[1,3], Mariya Pykhtina[1,3], Anatoly Beklemishev [1,3] Andrey Kovalskii[4], and Anastasiya Solovieva[1]

[1] Scientific Institute of Clinical and Experimental Lymphology– Branch of the ICG SB RAS, 2 Timakova str., 630060 Novosibirsk, Russian Federation, solovevaao@gmail.com (A.S.), ant-manahov@ya.ru (A.M.), svmiro@yandex.ru (S.M.), beklem@niibch.ru (AB), pykhtina_maria@mail.ru (MP), permyakova.elizaveta@gmail.com (E.P.

[2] Laboratory for the Physics of Advanced Materials (LPM), Department of Physics and Materials Science, University of Luxembourg, L-1511 Luxembourg, sergey.ershov@uni.lu (S.E.)

[3] Scientific Institute of Biochemistry, 2 Timakova str., 630060 Novosibirsk, Russian Federation, svmiro@yandex.ru (S.M.), beklem@niibch.ru (AB), pykhtina_maria@mail.ru (MP)

[4] National University of Science and Technology "MISiS", Leninsky pr. 4, Moscow 119049, Russia; andreykovalskii@gmail.com (A.K.)

* Correspondence: ant-manahov@ya.ru; Tel.: +7-915-8494059

1. Aminoacids sequence of grANG
2. Table S1 Calculation of the functional groups in the Apo-A1 using its chemical structure
3. Table S2 Calculation of the functional groups in the Angiogenin using its chemical structure
4. Measurement of APO-A1 concentration

1. Aminoacids sequence of hrANG

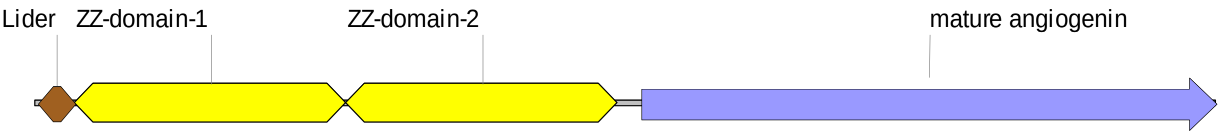

ZZ-hAngiogenin
252 aa

MAKIVQDVDNKFNKEQQNAFYEILHLPNLNEEQRNAFIQSLKDDPSQSANLLAEAKKL
NDAQAPKVDNKFNKEQQNAFYEILHLPNLNEEQRNAFIQSLKDDPSQSANLLAEAKK
LNDAQAPKVDANSMQDNSRYTHFLTQHYDAKPQGRDDRYCESIMRRRGLTSPCKDIN
TFIHGNKRSIKAICENKNGNPHRENLRISKSSFQVTTCKLHGGSPWPPCQYRATAGFRN
VVVACENGLPVHLDQSIFRRP

Figure S1. Scheme of a chimeric protein containing two Z-regions of protein A from Staphylococcus aureus and the amino acid sequence of mature human angiogenin. Each symbol reflect the aminoacid[1]

[1] - **The amino acids, symbols, and codons**
http://www.math.utep.edu/Faculty/mleung/bioinformatics/aacodon.html

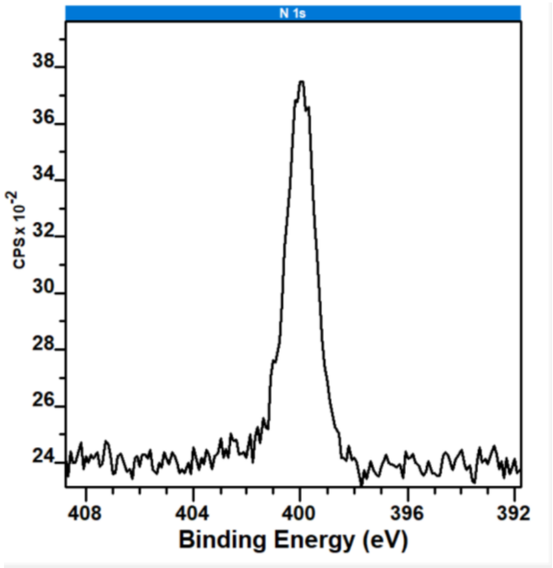

a)

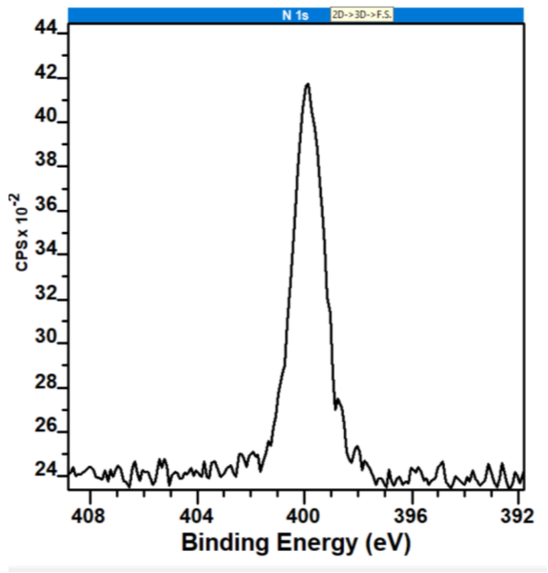

b)

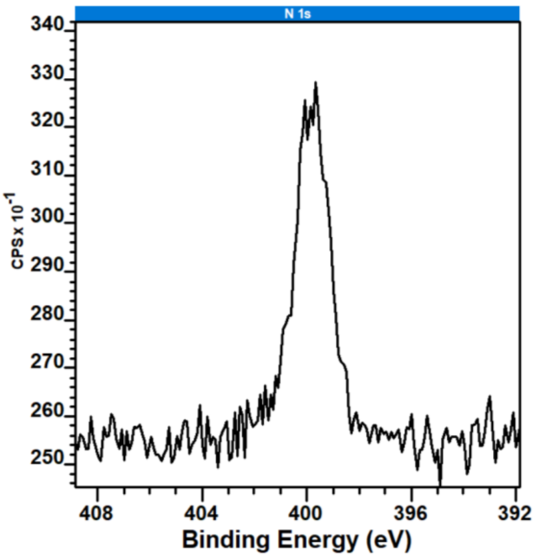

c)

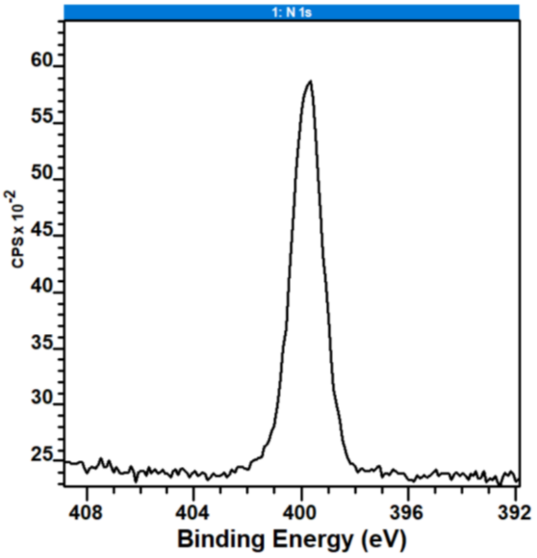

d)

Figure S2. XPS N1s curve fitting of PCL-Apo(a), PCL-COOH-Apo(b), PCL-COOH-ANG(c) and PCL-COOH-FN (d).

2. Table S1. Calculation of the functional groups in the Apo-A1 using its chemical structure

Aminoacid	Quantity	C=C	C-H	C-COOH	C=C-N	C-N	C-OH	C-S	N-C=O	N-C=N	COOH
Alanine	19	0	19	0	0	19	0	0	19	0	0
Serine	15	0	0	0	0	15	15	0	15	0	0
Aspartic acid	16	0	0	16	0	16	0	0	16	0	16
Arginine	16	0	32	0	0	32	0	0	16	16	0
Valine	12	0	48	0	0		0	0		0	0
Threonine	10	0	10	0	0	10	10	0	10	0	0
Glutamic acid	30	0	30	30	0	30	0	0	30	0	0
Leucine	37	0	148	0	0	37	0	0	37	0	0
Cysteine	0	0	0	0	0	0	0	0	0	0	0
Histidine	5	0	5	0	10	5	0	0	5	5	0
Lysine	21	0	63	0	0	42	0	0	21	0	0
Isoleucine	0	0	0	0	0	0	0	0	0	0	0
Tyrosine	7	35	7	0	0	7	7	0	7	0	0
Asparagine	5	0	0	5	0	5	0	0	10	0	0
Methionine	3	0	3	0	0	3	0	6	3	0	0
Proline	10	0	20	0	0	20	0	0	10	0	0
Tryptophan	4	28	4	0	4	4	0	0	4	0	0
Phenylalanine	6	36	6	0	0	6	0	0	6	0	0
Glutamine	16	0	16	16	0	16	0	0	32	0	0
Glycine	10	0	0	0	0	10	0	0	10	0	0
Total number		99	411	67	14	277	32	6	251	21	16
Concentration (%)		8.29	34.42	5.61	1.17	23.20	2.68	0.50	21.02	1.76	1.34

3. Table S2 Calculation of the functional groups in the Angiogenin using its chemical structure

**Aminoacid**	**Quantity**	**C=C**	**C-H**	**C-COOH**	**C=C-N**	**C-N**	**C-OH**	**C-S**	**N-C=O**	**N-C=N**	**COOH**
Alanine	6		6			6			6		
Serine	9					9	9		9		
Aspartic acid	6			6		6			6		6
Arginine	13		26			26			13	13	
Valine	9		36						9		
Threonine	9		9			9	9		9		
Glutamic acid	4		4	4		4			4		
Leucine	14		56			14			14		
Cysteine	6		6			6		6	6		
Histidine	6		6		12	6			6	6	
Lysine	7		21			14			7		
Isoleucine	7		28			7			7		
Tyrosine	4	20	4			4	4		4		
Asparagine	9			9		9			18		
Methionine	3		3			3		6	3		
Proline	10		20			20			10		
Tryptophan	1	7	1		1	1			1		
Phenylalanine	6	36	6			6			6		
Glutamine	6		6	6		6			12		
Glycine	12		12			12			12		
Total number		63	250	25	13	168	22	12	162	19	6
Concentration (%)		8.51	33.8	3.38	1.76	22.70	2.97	1.62	21.89	2.57	0.81

4. Measurement of APO-A1 concentration

The concentration of APO-A1 was performed by measuring the fluorescence in the PBS after washing the sample. In order to calculate the concentration of APO-A1 from the fluorescence intensity, a calibration curve for standard solution of APO-A1 was obtained. The concentration of APO-A1 in PBS was varied from 500 to 0.1 µg/mL. The fluorescence signal was measured was each solution and summarized in Figure S1. The exponential data was fitted using exponential function and the Equation S1 was used for estimation of the APO-A1 concentration for the PCL-APO and PCL-COOH-APO samples after soaking in PBS for 20 min, 48h and 144 h.

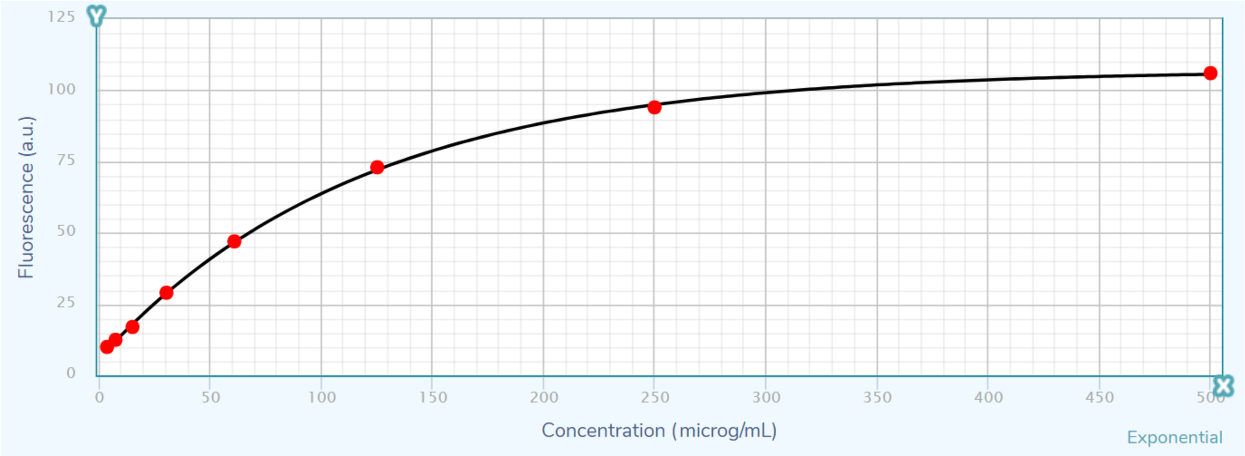

Figure S3. Calibration curve for APO-A1 concentration measurement.

$$y = 6.045386 - (-0.8574563/0.008488019) * (1 - e\hat{}(-0.008488019 * x)) \quad \textit{Equation S1}$$
